# GLORIOUS PAST, BRIGHTER FUTURE

**DOI:** 10.4103/0019-5154.40563

**Published:** 2008

**Authors:** Sandipan Dhar

**Affiliations:** *Form the Pediatric Dermatology Division, Institute of Child Health, Kolkata, India*

The year 2007 has been very hectic and eventful for the Indian Journal of Dermatology (IJD). This was the 52nd year of this journal. The web version of the journal www.e-ijd.org has always been quite popular and there has been a steady increase in the number of contributions to the journal over the last couple of years. In 2007, we had a record 195 submissions, of which 56 were original articles and 12 were review articles. The policy of the journal has been to invite Review and CME articles from academicians who have worked in some particular field for number of years; we have been overwhelmed by the growing number of good quality review articles being uploaded to IJD's website by various contributors. This enthusiastic response has encouraged us a lot in our efforts to make the journal better and richer in its content.

The rejection rate of a journal reflects the quality control being exercised by the editorial staff with regard to its scientific material. The rejection rate of IJD rose to 32% in the year 2007. The figure is comparable with that of some of the most prestigious international journals devoted to the subject of dermatology, venereology and leprology.

Ever since making its appearance on the Internet as the first journal of dermatology from India, IJD has enjoyed a steady increase in its popularity, as reflected by the number of hits on the site and its rising impact factor. A Google search shows that IJD ranks 5^th^ among all Indian medical journals with Web presence. This is a quantum leap forward and a jump of seven ranks from 12^th^ position last year. In the last few issues, in our section on basic research, we have published some fine research work of high scientific value. We are grateful to the contributors and look forward to their continued support and many more similar contributions.

I must thank my two most able colleagues, both executive editors, Dr. Koushik Lahiri and Dr. Saumya Panda for their tireless efforts to make the journal perfect in all possible ways! I also thank all the members of my hard-working editorial team, the committed editorial board members, sincere reviewers, committed contributors, and above all my ‘vote bank’—the selfless, unbiased readers! The journal strives for perfection because of its readers; I don't ever dare forget that.

Currently it takes about 6 to 7 months for an original contribution, and 12 to 18 months for a case report, to be published. We have been working hard to shorten the duration so as to make the journal more contributor / author friendly. We have been regularly publishing dermatology-related articles from different specialties in our journal. This, we know, makes the journal more interesting for its readers. In the next few issues we will be publishing some of the finest of such articles and readers can look forward to that.

I take this opportunity to thank Dr. D K Sahu of Medknow Publications for his help and support in improving the quality of the journal. His perseverance was instrumental in giving IJD its 5^th^ rank. Maintaining the standard of the journal is only possible if we feel proud of our journal and are happy to publish our best work in it. Authors should realize that by publishing their work in an Indian journal they are better able to communicate with their colleagues in the country as they are more accessible to them. We need to feel proud to publish articles in Indian journals.

A journal can never run smoothly without funding from the pharmaceutical industry. I must thank GlaxoSmithKline (Derma), Galderma India, Fulford India (Ltd), Stiefel India (Pvt) Ltd., Gracewell Derma, and Ajanta Pharma who have supplied the necessary oxygen for this journal's viability.

I, on behalf of the ‘Team IJD,’ sincerely thank all of you for your kind patronage of IJD and wish you a very happy, fruitful, and prosperous 2008.

**Sandipan Dhar**

Editor- IJD

